# Predictors for delayed awakening in adult glioma patients receiving awake craniotomy under monitored anesthesia care

**DOI:** 10.1007/s11060-023-04494-1

**Published:** 2023-11-02

**Authors:** Huan-Tang Lin, Chun-Ming Lin, Yah-Yuan Wu, Wei-Han Chang, Kuo-Chen Wei, Yi-Chun Chen, Pin-Yuan Chen, Fu-Chao Liu, Ko-Ting Chen

**Affiliations:** 1 Department of Anesthesiology, College of Medicine, Chang Gung Memorial Hospital at Linkou, Chang Gung University, Taoyuan, 333 Taiwan; 2 Graduate Institute of Clinical Medical Sciences, College of Medicine, Chang Gung University, Taoyuan, 333 Taiwan; 3 Department of Neurology, College of Medicine, Chang Gung Memorial Hospital at Linkou, Chang Gung University, Taoyuan, 333 Taiwan; 4 Department of Physical Medicine & Rehabilitation, College of Medicine, Chang Gung Memorial Hospital at Linkou, Chang Gung University, Taoyuan, 333 Taiwan; 5 Department of Neurosurgery, College of Medicine, Chang Gung Memorial Hospital at Linkou, Chang Gung University, 5 Fu-Shin Street, Kwei-Shan, Taoyuan, 333 Taiwan; 6 Neuroscience Research Center, Chang Gung Memorial Hospital at Linkou, Taoyuan, 333 Taiwan

**Keywords:** Awake craniotomy, Glioma, Somnolence, Isocitrate dehydrogenase, Karnofsky performance scale

## Abstract

**Purpose:**

Delayed awakening after anesthetic discontinuation during awake craniotomy is associated with somnolence during functional brain mapping. However, predictors of delayed awakening in patients receiving monitored anesthesia care for awake craniotomy are unknown.

**Methods:**

This retrospective cohort study analyzed 117 adult patients with supratentorial glioma in or near eloquent areas who received monitored anesthesia care for awake craniotomy between July 2020 and January 2023 at Linkou Chang Gung Memorial Hospital. These patients were divided into two groups according to their time to awakening (ability to speak their names) after propofol cessation: longer or shorter than 20 min (median duration). Because propofol was solely used anesthetic from skin incision to dural opening, parameters in Schnider model for propofol target-controlled infusion, such as age, sex, and BMI, were adjusted or propensity-matched to compare their anesthetic, surgical, and histopathological profiles.

**Results:**

After propensity-matched comparisons of age and BMI, significant predictors of delayed awakening included IDH1 wild-type tumors and repeated craniotomies. Subgroup analysis revealed that older age and larger T2 volume were predictors in patients undergoing the first craniotomy, while lower preoperative Karnofsky performance scale scores and depression were predictors in repeated craniotomy cases. Delayed awakening was also associated with somnolence and a lower gross total resection rate.

**Conclusion:**

Our retrospective analysis of patients receiving monitored anesthesia care for awake craniotomy revealed that delayed awakening after propofol discontinuation occurred more often in patients with IDH1 wild-type tumors and repeated craniotomies. Also, delayed awakening was associated with somnolence during functional mapping and a lower gross total resection rate.

**Supplementary Information:**

The online version contains supplementary material available at 10.1007/s11060-023-04494-1.

## Introduction

Awake craniotomy is a well-established approach for resecting brain tumors in or near the eloquent cortex. This approach aims to maximize patient safety while preserving postoperative neurological functions [1]. During awake craniotomy, intraoperative functional mapping of the eloquent cortex and subcortical pathways is an important step to evaluate the functionality of the underlying brain tissues and avoid damage to potential functional areas [2]. Awake mapping has significantly reduced postoperative morbidities and improve the extent of tumor resection. Aggressive resection guided by functional mapping may provide a survival benefit in both primary and recurrent brain tumors [2–4]. With appropriate preoperative preparation and intraoperative management, awake craniotomy can be safely performed in various patient types, minimizing discomfort and achieving low complication rates [1, 2]. A recent meta-analysis revealed that awake craniotomy was associated with a higher extent of tumor resection, lower postoperative neurological and language deficits, less postoperative nausea and vomiting, decreased morbidity, shorter length of hospital stay, and faster recovery compared to craniotomy under general anesthesia [5].

Generally, conscious sedation techniques in awake craniotomy are classified as the monitored anesthesia care (MAC) technique using an unprotected airway or the asleep-awake-asleep (SAS) technique using a laryngeal mask airway or endotracheal intubation [6, 7]. A retrospective analysis of 225 patients in China found that the MAC technique was associated with shorter operative duration and less postoperative dysfunction than the SAS technique [8]. A recent meta-analysis found that the MAC technique was associated with a lower failure rate, shorter surgical duration, and a lower incidence of nausea and vomiting than the SAS technique [6]. The anesthetic regimen for the MAC technique varies among different countries and surgical teams but generally consists of a combination of propofol/remifentanil or propofol/remifentanil/dexmedetomidine, often with the addition of a scalp block [9].

Delayed awakening during awake craniotomy could compromise the accuracy of functional mapping. Patients experiencing prolonged emergence are at risk of developing adverse events such as somnolence, seizure, vomiting, or coughing during emergence [10]. A retrospective Japanese cohort study of 55 patients reported preoperative aphasia as a predictor of somnolence during mapping using the SAS technique [11]. Another retrospective Dutch cohort study found that older age, non-smoking status, and having an American Society of Anesthesiologists (ASA) classification of III were associated with delayed wakefulness exceeding 20 min after the cessation of propofol and remifentanil in SAS for awake craniotomy [10]. At our institute, we have been using the MAC technique, which combines propofol infusion with a scalp block, for awake craniotomy with a high success rate for decades. However, the predictors of delayed awakening with this technique have never been discussed, and the differences in anesthetic and surgical profiles between first and repeated awake craniotomies have never been compared in the literature. Identifying the predictors of delayed awakening could help us identify susceptible patients in advance and adjust anesthetic regimens accordingly. Therefore, this study aimed to retrospectively identify possible predictors of delayed awakening after propofol discontinuation during MAC for awake craniotomy.

## Materials and methods

This retrospective, single-center study aimed to evaluate the predictors of prolonged awakening in adult patients who underwent the MAC technique for awake craniotomy. This study was permitted by the Institutional Review Board of the Chang Gung Medical Foundation, Taiwan (approval number: 202201692B0). Due to the retrospective nature and untraceable personal information in the study, the requirement for written informed consent was waived.

### Study population

**Fig. 1:**
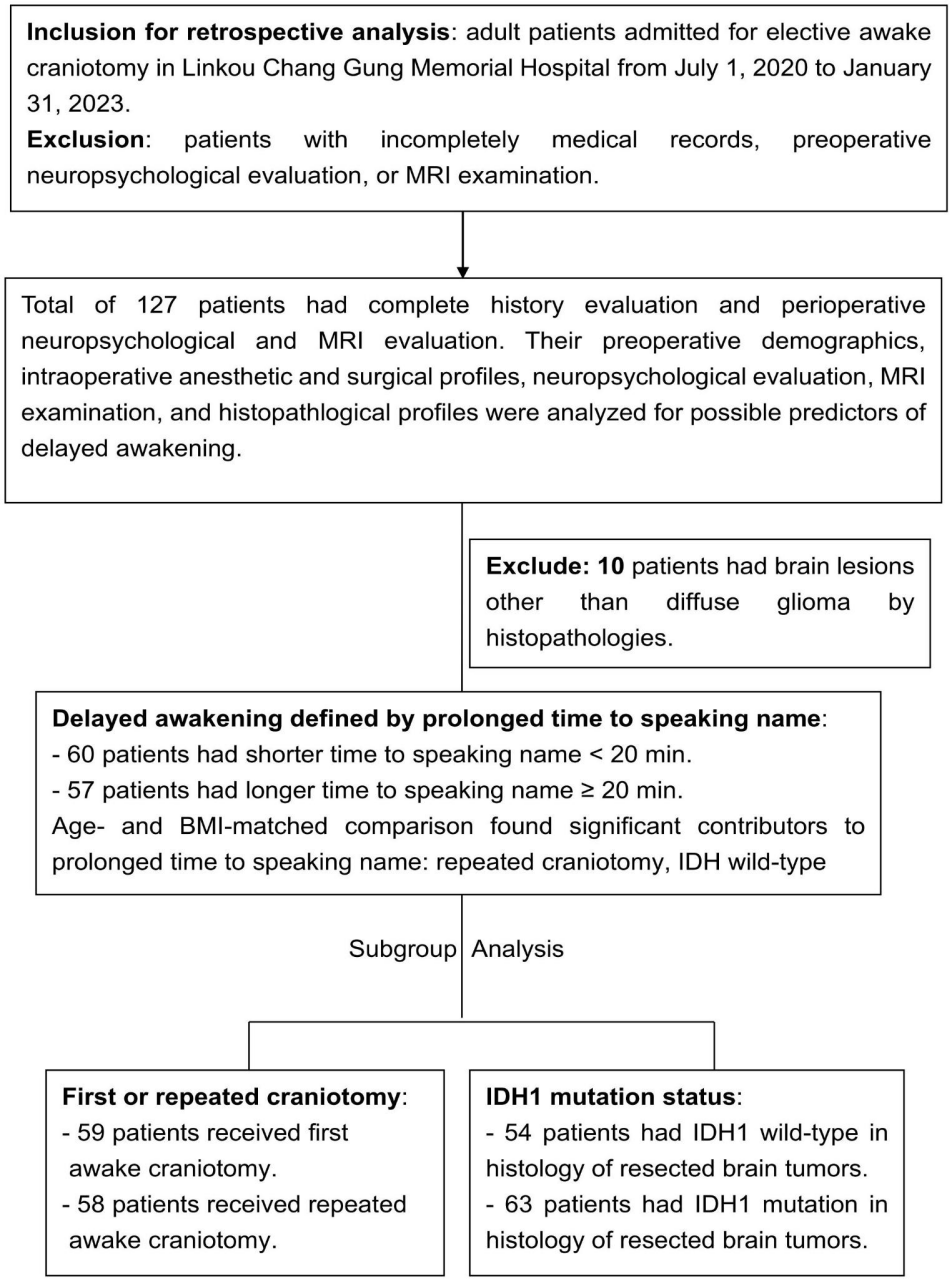
Flow chart for the study design and group separation

The inclusion criteria consisted of adult patients who (1) had a pathology-proven glioma (World Health Organization (WHO) grade I to IV) located in or near the cerebral eloquent cortex and (2) underwent awake craniotomy using the MAC technique at Linkou Chang Gung Memorial Hospital. Patients with non-glioma pathologies, incomplete medical records, or incomplete preoperative neuropsychological evaluation were excluded from the study [12]. From July 1, 2020, to January 31, 2023, 117 adult patients were enrolled in the analysis (Fig. 1). Each participant received T1-weighted magnetic resonance imaging (MRI) after contrast enhancement, and T2 fluid-attenuated inversion recovery (FLAIR) imaging for volumetric analysis before and after surgical resection. The extent of resection was determined by T1 contrast-enhancing volume for WHO grade IV gliomas and by T2/FLAIR volume for low grade gliomas (WHO grades II and III) [13]. Furthermore, the extent of resection was classified as gross total resection (GTR) (95–100%), subtotal resection (85–95%), or partial resection (< 85%), according to the classification described by Sawaya et al. [14]. Tumor location was divided into frontal, parietal, temporal, corpus callosum, insular, and hippocampal regions. For tumors that invaded more than one lobe, only the major involved lobes were counted. The histopathological diagnoses were all determined by a senior neuropathologist, and the grading criteria of gliomas were based on the WHO 2021 classification system [15]. The IDH1 mutation status was determined by immunohistochemistry using an anti-R132H-IDH1 antibody. The first craniotomy was defined as the initial awake surgery for newly diagnosed brain tumors, and repeated craniotomy referred to a redo awake surgery for recurrent tumors in or near the eloquent cortex following a prior awake or anesthetic surgery.

### Awake craniotomy preparation and monitored anesthesia care

The MAC technique with propofol sedation was administered to all patients as the main anesthesia regimen. Initially, moderate sedation was achieved using propofol infusion accompanied by an intermittent bolus of midazolam or fentanyl at the discretion of duty anesthesiologist. Following sedation, invasive hemodynamic monitoring, including arterial line and central venous catheterization, was performed, and a urinary catheter was inserted. A scalp block was administered using either 20 ml of 0.5% levobupivacaine (Chirocaine, Abbott) or 20 ml of 1% xylocaine (Lidocaine, LITA Pharmacy Co.). Intravenous parecoxib 40 mg (Dynastat, Pfizer) was given for multimodal analgesia. Besides, local anesthetics with epinephrine was infiltrated at the headpin sites and the planned incision line. Dexamethasone (5 mg; Methasone; Taiwan Biotech Co.) and ondansetron (8 mg; Supren; Taiwan Biotech Co.) were intravenously administered to prevent nausea and vomiting. Subsequently, the patients were positioned either supine or semilateral, depending on the location of the glioma, with the head fixed in a Mayfield holder. Standard neuro-navigation was applied for craniotomy guidance with Medtronic Stealth S7 system (Medtronic®).

Adequate airway patency was maintained using a suitable nasal airway with the tip above the glottis, supplying oxygen at a flow rate of 6 L/min via a simple mask. Adequate gas exchange was confirmed through arterial blood gas analysis, showing fair oxygenation and normocapnia. Propofol was the sole sedative used from skin incision to dural opening (turning off), administered in an effect-site target-controlled infusion pump (Schnider model). Dexmedetomidine and remifentanil were not utilized in our MAC technique because these drugs were not covered by health insurance or unavailable in our institute during the study period. The effect-site concentration (Ce) of propofol was titrated between 2.0 and 2.2 µg/mL to maintain moderate sedation, assessed using the modified Observer’s Assessment of Alertness/Sedation Scale (OAA/S) score 0–2 or Bispectral index (BIS) 60–80 if used [16, 17]. After discontinuing propofol at dural opening, the patients were prompted every 30 s to awaken by opening their eyes or speaking their names. The amount of propofol infused before cessation was recorded as the total dose (mg). The estimated propofol Ce at the time point of “turning off,” responsive to “opening eyes,” “speaking names,” and “beginning of functional mapping,” were recorded. Since speaking is crucial for functional mapping and indicates subcortical-cortical connectivity [18], we defined the time from propofol cessation to speaking one’s name as “time to awakening.” Because anesthesia depth monitoring is not universally covered by health insurance in Taiwan, the BIS value was not recorded for every participant and was not analyzed in this cohort. After the patients became alert and could perform functional testing, experienced neuropsychologists continuously assessed their awake language and motor functions throughout the whole tumor resection process, which typically lasted 1.5–2.5 h. Following maximal tumor resection, propofol infusion was resumed to maintain sedation until surgery completion. Subsequently, patients were transferred to a neurosurgical intensive care unit (ICU) for postoperative care.

### Statistical analysis

The primary outcome was the contributing factors to delayed awakening after discontinuation of propofol. We defined delayed awakening as a time to speaking one’s name ≥ 20 min for two reasons: the median time to speaking name in our cohort was 19 min, and a previous Dutch cohort also used 20 min as the threshold for prolonged awakening [10]. The secondary outcome was the comparison of different predictors of delayed awakening in patients receiving the first or repeated craniotomy and in patients with IDH1 mutant or wild-type gliomas, as these factors are significant contributors to prolonged awakening in age- and BMI-matched comparisons (Table 1). Enrolled patients’ demographics including age, sex, Karnofsky performance scale (KPS) scores, neuropsychological evaluations, tumor characteristics, anesthetic and surgical profiles, histopathological examinations, and tumor resection rates were compared. Data are expressed as mean ± standard deviation for continuous variables and percentages for qualitative variables (sex and tumor characteristics). The statistical analyses utilized Student’s t-test or χ^2^ tests for comparison between two-groups and analysis of variance (ANOVA) for comparison between multiple groups. Because age, sex, weight, and height are required parameters in Schnider model of propofol target-controlled infusion pump [19], age and BMI 1:1 propensity score-matched comparisons or adjustments for age, sex, and BMI were performed during comparison to eliminate demographic differences. SAS software (version 9.4; SAS Institute, Inc. NC, USA) was utilized for all statistical analyses, and a two-sided *p* value < 0.05 was considered statistically significant.

## Results

### Patient groups and their demographic comparison

Figure 1 illustrates the grouping method used in this study. Based on the median time to speaking one’s name in our cohort of 19 (interquartile range, 14–26) min, we divided our cohort into two groups: those with a time to speaking a name shorter and longer than 20 min (defined as faster or delayed awakening, respectively). Supplementary Table 1 presents the demographic comparisons between the two groups, showing that patients with a prolonged awakening had lower preoperative KPS, more history of craniotomies, higher WHO grade, lower propofol concentration while awake, and lower OAA/S scores during functional mapping. To eliminate potential confounding factors related to age and BMI [19], we conducted age and BMI 1:1 propensity-matched comparisons between the two groups, and the results are listed in Table 1. Following these comparisons, patients with a prolonged awakening had a significantly higher history of craniotomies and fewer IDH1 mutations than patients with a faster awakening. Therefore, repeated craniotomy and IDH1 wild-type tumors were identified as important predictors of delayed awakening. Subsequently, we analyzed these factors to further explore the factors contributing to delayed awakening in different patient groups.

**Table 1:** Age- and BMI-matched comparison between patients with diverse time to speaking name

Variables	Time to speaking name < 20 min (n = 42)	Time to speaking name ≥ 20 min (n = 42)	*p* ^#^	OR
Age (mean ± SD, years)	49.97 ± 16.43	50.52 ± 13.96	0.869	0.98
Male sex, N(%)	21 (50.00%)	20 (47.62%)	0.827	0.90
BMI (kg/m^2^)	24.33 ± 3.95	24.46 ± 3.22	0.869	0.97
Preoperative creatinine (mg/dL)	0.71 ± 0.19	0.70 ± 0.17	0.739	0.96
Preoperative total bilirubin(mg/dL)	0.55 ± 0.26	0.59 ± 0.21	0.780	1.96
Preoperative hypnotics use	2 (4.87%)	2 (4.87%)	1.000	0.97
Preoperative KPS score	87.43 ± 9.38	85.00 ± 9.61	0.257	0.97
Hypertension	12 (28.57%)	8 (19.04%)	0.305	0.58
Diabetes	4 (9.52%)	2 (4.76%)	0.675	0.48
Old stroke	1 (2.38%)	2 (4.76%)	0.675	2.04
smoking	6 (14.28%)	3 (7.14%)	0.482	0.46
History of brain tumor surgery	14 (33.33%)	24 (57.14%)	0.028*	2.66*
Neuropsychological evaluation				
Aphasia	11 (26.19%)	12 (28.57%)	0.931	1.05
Depression	7 (17.95%)	10 (27.02%)	0.342	1.69
Executive dysfunction	11 (28.20%)	14 (37.83%)	0.371	1.54
Brain tumor characters				
Left hemisphere	27 (64.28%)	25 (59.52%)	0.824	1.00
Frontal lobe	24 (57.14%)	22 (52.38%)	0.921	1.00
Temporal lobe	10 (23.81%)	13 (30.95%)	0.921	0.61
Parietal lobe	4 (9.52%)	3 (7.14%)	0.921	1.41
Corpus callosum	13 (30.95%)	8 (19.05%)	0.921	0.52
Insula	3 (7.14%)	3 (7.14%)	0.921	1.09
Hippocampus	1 (2.38%)	0 (0%)	0.921	NA
T1 enhanced volume (ml)	14.39 ± 21.13	16.21 ± 25.53	0.723	1.01
T2/FLAIR volume (ml)	84.03 ± 73.41	81.88 ± 56.96	0.881	0.99
Midline shift(mm)	3.57 ± 4.68	2.96 ± 4.16	0.537	0.98
WHO grade	3.07 ± 1.02	3.24 ± 0.72	0.392	1.24
IDH1 mutation	25 (59.52%)	13 (30.95%)	0.042*	0.56*
Propofol Ce (opening eyes) (µg/mL)	0.77 ± 0.21	0.60 ± 0.27	0.002*	0.07
Propofol Ce (speaking name) (µg/mL)	0.69 ± 0.19	0.51 ± 0.17	0.001*	0.01*
Propofol Ce (mapping) (µg/mL)	0.50 ± 0.17	0.42 ± 0.17	0.033*	0.01*
Propofol dose before cessation (mg)	614.47 ± 126.48	603.77 ± 203.78	0.777	0.99
Time to opening eye (min)	11.69 ± 3.72	21.26 ± 7.56	< 0.001*	1.50*
Time to speaking name (min)	14.40 ± 3.52	27.02 ± 7.11	< 0.001*	1.47*
Time to functional mapping (min)	25.71 ± 6.90	37.07 ± 9.13	< 0.001*	1.25*
OAA/S score before mapping	4.75 ± 0.49	4.37 ± 1.03	0.042*	0.53*
Somnolence	1 (2.50%)	8 (20.00%)	0.046*	0.13*

Abbreviation: OR, odds ratio; BMI, body mass index; IDH, isocitrate dehydrogenase; KPS, Karnofsky performance scale; FLAIR, fluid-attenuated inversion recovery; Ce, effect-site concentration; OAA/S score, Observer Assessment of Alertness/Sedation Scale; NA, non-available. ^#^*p* value was calculated using the chi-square test for categorical variables and the analysis of variance (ANOVA) for continuous variables. *p < 0.05

### Comparison between patients receiving first vs. repeated craniotomies

There were fifty-nine patients receiving their first craniotomy, whereas 58 underwent repeated craniotomies (Supplementary Table 2). A comparison of the potential contributors to delayed awakening in each group is presented in Table 2. In patients undergoing their first craniotomy, older age and larger glioma volume on MRI T2/FLAIR images were significant predictors of prolonged awakening. However, in patients who underwent repeated craniotomies, lower preoperative KPS and depression were significant contributors to prolonged awakening.

**Table 2:** Comparison of predictors for prolonged time to speaking one’s name in first or repeated brain tumor surgery

Variables	First brain tumor surgery (n = 59)				Repeated brain tumor surgery (n = 58)			
	**Time to speaking name < 20 min(n = 38)**	**Time to speaking name ≥ 20 min(n = 21)**	***p*** ^#^	**Adjusted OR** ^**#**^	**Time to speaking name < 20 min(n = 22)**	**Time to speaking name ≥ 20 min(n = 36)**	***p*** ^#^	**Adjusted OR** ^**#**^
Age (mean ± SD, years)	47.84 ± 16.87	60.52 ± 13.52	0.004*	1.05*	47.59 ± 12.72	47.36 ± 11.61	0.944	0.99
Male sex, N(%)	18(47.36%)	6(28.57%)	0.159	0.44	15(68.18%)	19(52.78%)	0.248	0.52
BMI (kg/m^2^)	25.39 ± 4.54	24.19 ± 3.30	0.291	0.93	25.52 ± 4.19	23.74 ± 3.28	0.077	0.87
Preoperative creatinine (mg/dL)	0.72 ± 0.21	0.66 ± 0.16	0.183	0.59	0.72 ± 0.15	0.70 ± 0.19	0.714	0.58
Preoperative hypnotics use	3(8.11%)	1(4.76%)	0.796	0.56	2(9.09%)	2(5.55%)	0.628	0.58
Preoperative KPS score	87.50 ± 7.69	84.28 ± 8.70	0.153	0.96	90.26 ± 10.76	80.36 ± 12.47	0.018*	0.95*
Hypertension	9(23.68%)	3(14.28%)	0.509	0.53	8(36.36%)	7(19.44%)	0.153	0.42
Diabetes	3(7.89%)	1(4.76%)	0.867	0.62	2(9.09%)	2(5.56%)	0.629	0.59
Old stroke	1(2.63%)	1(4.76%)	0.762	1.85	0(0%)	2(5.56%)	0.521	NA
Neuropsychological evaluation								
Aphasia	11(30.55%)	6(30.00%)	0.965	0.97	4(18.18%)	11(30.55%)	0.191	2.35
Depression	10(27.77%)	4(19.05%)	0.519	0.65	2(9.52%)	12(37.50%)	0.024*	5.69*
Executive dysfunction	11(30.55%)	8(38.09%)	0.474	1.26	5(23.81%)	14(43.75%)	0.138	2.48
Brain tumor characters								
Left hemisphere	23(60.52%)	14(66.67%)	0.641	1.10	13(59.09%)	24(66.67%)	0.891	1.00
Right hemisphere	15(39.47%)	7(33.33%)	0.641	0.92	9(40.91%)	12(33.33%)	0.891	0.86
Frontal lobe	24(63.15%)	10(47.62%)	0.643	1.00	14(63.63%)	22(61.11%)	0.862	1.00
Temporal lobe	9(23.68)	7(33.33%)	0.643	1.07	4(18.18%)	7(19.44%)	0.862	0.745
Parietal lobe	3(7.89%)	3(14.29%)	0.643	4.50	2(9.09%)	3(8.33%)	0.862	1.807
Corpus callosum	12(31.58%)	6(28.57%)	0.643	0.87	6(27.27%)	6(16.67%)	0.862	0.533
Insula	1 (2.63%)	1 (4.76%)	0.643	2.42	2 (9.09%)	2 (5.55%)	0.862	1.24
Hippocampus	1 (2.63%)	0 (0%)	0.643	NA	0	0	0.862	NA
T1 enhanced volume (ml)	14.85 ± 24.28	22.15 ± 27.00	0.300	1.48	10.75 ± 18.01	13.73 ± 23.08	0.608	1.01
T2/FLAIR volume (ml)	72.80 ± 72.57	109.62 ± 44.93	0.042*	1.49*	72.64 ± 75.19	71.10 ± 66.23	0.935	0.99
Midline shift(mm)	4.08 ± 4.62	7.77 ± 12.17	0.195	1.07	1.94 ± 3.12	2.41 ± 4.37	0.639	1.07
WHO grade	3.05 ± 1.01	3.42 ± 0.67	0.133	1.63	3.04 ± 0.89	3.22 ± 0.76	0.426	1.31
IDH1 mutation	17(45.94%)	7(33.33%)	0.348	0.73	10(45.45%)	19(52.78%)	0.588	1.34
Propofol Ce (opening eyes) (µg/mL)	0.78 ± 0.21	0.47 ± 0.22	0.001*	0.62*	0.81 ± 0.17	0.62 ± 0.26	0.003*	0.03*
Propofol Ce (speaking name) (µg/mL)	0.69 ± 0.19	0.41 ± 0.16	0.001*	0.59*	0.77 ± 0.18	0.52 ± 0.16	0.001*	0.01*
Propofol Ce (mapping) (µg/mL)	0.49 ± 0.17	0.34 ± 0.16	0.001*	0.69*	0.53 ± 0.13	0.43 ± 0.16	0.019*	0.01*
Propofol dose before cessation (mg)	603.46 ± 107.27	551.41 ± 151.53	0.192	0.99	702.43 ± 193.13	633.54 ± 197.19	0.201	0.99
Time to opening eye (min)	11.92 ± 3.60	23.33 ± 8.79	0.001*	1.59*	10.77 ± 3.36	22.05 ± 6.84	0.001*	1.58*
Time to speaking name (min)	14.34 ± 3.40	28.72 ± 8.52	0.001*	1.34*	13.27 ± 3.73	28.61 ± 7.25	0.001*	1.34*
Time to functional mapping (min)	26.42 ± 6.95	40.19.±11.73	0.001*	1.22*	26.59 ± 9.31	38.11 ± 8.86	0.001*	1.20*
OAA/S score before mapping	4.81 ± 0.46	4.28 ± 1.00	0.033*	0.43*	4.81 ± 0.40	4.35 ± 0.95	0.017*	0.23*

Abbreviation: OR, odds ratio; BMI, body mass index; IDH, isocitrate dehydrogenase; KPS, Karnofsky performance scale; FLAIR, fluid-attenuated inversion recovery; Ce, effect-site concentration; OAA/S score, Observer Assessment of Alertness/Sedation Scale; NA, non-available. ^#^*p* value was calculated using the chi-square test for categorical variables and the analysis of variance (ANOVA) for continuous variables. Adjusted OR was calculated by adjusted with age, sex, and BMI. *p < 0.05

### Comparison between patients with IDH1 mutant vs. wild-type gliomas

Demographic comparisons between patients with IDH1 mutant and wild-type gliomas are shown in supplementary Table 3. After adjusting for age, sex, and BMI, patients with IDH1 wild-type tumors had significantly higher T1 enhanced volume, WHO grade, and Ki-67% compared to patients with IDH1 mutant tumors. The correlation map in Supplementary Fig. 1 compares the correlations between IDH1 mutation status, radiographic tumor volume (T1, T2/FLAIR images, and midline shift), and time to speaking one’s name. It showed that the time to speaking one’s name had a significantly positive correlation with radiographic midline shift in both IDH1 wild-type (*r* = 0.37, *p* < 0.05) and mutant tumors (*r* = 0.42, *p* < 0.05). Table 3 presents a further comparison of predictors of prolonged time to speaking names in patients with IDH1 mutations and wild-type tumors. In patients with IDH1 mutations, higher WHO grade and previous craniotomy significantly contributed to prolonged awakening.

**Table 3:** Comparison of predictors for prolonged time to speaking one’s name in patients with IDH mutant or wild-type tumors

Variables	IDH1 mutation (n = 54)				IDH1 wild-type (n = 63)			
	Time to speaking name < 20 min(n = 27)	Time to speaking name ≥ 20 min(n = 27)	*p* ^#^	Adjusted OR^#^	Time to speaking name < 20 min(n = 32)	Time to speaking name ≥ 20 min(n = 31)	*p* ^#^	Adjusted OR^#^
Age (mean ± SD, years)	39.67 ± 11.03	46.27 ± 11.36	0.037*	1.21*	54.81 ± 15.39	57.19 ± 13.85	0.521	1.01
Male sex, N(%)	15 (55.55%)	12 (46.15%)	0.493	0.78	17 (53.13%)	13 (41.93%)	0.374	0.63
BMI (kg/m^2^)	25.54 ± 3.95	25.41 ± 3.12	0.892	0.98	25.12 ± 4.63	22.65 ± 2.86	0.013*	0.81*
Preoperative creatinine (mg/dL)	0.72 ± 0.19	0.71 ± 0.17	0.746	1.04	0.72 ± 0.18	0.67 ± 0.19	0.306	0.93
Preoperative hypnotics use	2 (7.69%)	1 (3.84%)	0.487	0.48	3 (9.37%)	2 (6.45%)	0.607	0.67
Preoperative KPS score	90.80 ± 7.59	86.25 ± 10.13	0.081	0.95	85.81 ± 9.58	81.33 ± 11.05	0.096	0.95
Hypertension	6 (22.22%)	3 (11.54%)	0.467	0.47	10 (31.25%)	7 (22.58%)	0.438	0.64
Diabetes	0 (0%)	1 (3.85%)	0.491	NA	4 (12.50%)	2 (6.45%)	0.672	0.48
Old stroke	0 (0%)	1 (3.84%)	0.491	NA	1 (3.13%)	2 (6.45%)	0.613	2.13
Previous craniotomy	10 (37.04%)	19 (73.07%)	0.008*	2.02*	12 (37.50%)	17 (54.83%)	0.167	2.02
Neuropsychological evaluation								
Aphasia	5 (18.52%)	6 (24.00%)	0.628	1.39	10 (33.33%)	11 (40.74%)	0.563	1.37
Depression	5 (18.52%)	8 (32.00%)	0.262	2.07	7 (24.13%)	8 (29.63%)	0.643	1.32
Executive dysfunction	5 (18.52%)	10 (40.00%)	0.087	2.93	10 (34.48%)	12 (44.44%)	0.446	1.51
Brain tumor characters								
Left hemisphere	16 (59.24%)	16 (61.54%)	0.573	1.00	19 (59.37%)	22 (70.96%)	0.335	1.00
Right hemisphere	10 (37.04%)	9 (34.61%)	0.573	0.78	13 (40.63%)	9 (29.04%	0.335	1.07
Frontal lobe	23 (85.00%)	17 (65.38%)	0.441	1.00	15 (46.87%	15 (48.38%)	0.676	1.00
Temporal lobe	3 (11.11%)	5 (19.23%)	0.441	0.32	10 (31.25%	9 (29.03%)	0.676	0.64
Parietal lobe	0 (0%)	1 (3.85%)	0.441	0.21	4 (12.50%)	5 (16.13%)	0.676	0.27
Corpus callosum	11 (40.74%)	6 (23.08%)	0.441	0.55	7 (21.87%)	6 (19.35%)	0.676	0.86
Insula	1 (3.70%)	2 (7.69%)	0.441	2.71	2 (6.25%)	1 (3.23%))	0.676	0.02
Hippocampus	0 (0%)	0 (0%)	0.441	NA	1 (3.13%)	0 (0%)	0.676	NA
T1 enhanced volume (ml)	6.79 ± 20.71	10.01 ± 25.26	0.613	1.07	17.99 ± 22.03	22.55 ± 22.95	0.428	1.01
T2/FLAIR volume (ml)	71.63 ± 61.82	81.72 ± 65.24	0.565	1.59	84.89 ± 79.15	82.20 ± 57.76	0.878	0.99
Midline shift(mm)	3.06 ± 4.14	3.41 ± 4.51	0.771	1.58	3.70 ± 4.96	4.70 ± 10.37	0.631	1.02
WHO grade	2.55 ± 0.64	2.92 ± 0.56	0.031*	2.81*	3.46 ± 1.05	3.61 ± 0.71	0.518	1.21
Ki-67(%)	16.60 ± 18.77	13.14 ± 11.88	0.488	0.98	26.16 ± 27.63	23.56 ± 15.74	0.685	0.99
Jafari classification	1.81 ± 0.98	1.92 ± 1.01	0.678	0.97	2.21 ± 1.00	2.48 ± 0.96	0.289	1.56
Propofol Ce (opening eyes) (µg/mL)	0.84 ± 0.16	0.65 ± 0.29	0.004*	0.80*	0.74 ± 0.21	0.49 ± 0.19	0.001*	0.61*
Propofol Ce (speaking name) (µg/mL)	0.79 ± 0.17	0.52 ± 0.18	0.001*	0.72*	0.67 ± 0.19	0.44 ± 0.15	0.001*	0.68*
Propofol Ce (mapping) (µg/mL)	0.55 ± 0.14	0.43 ± 0.18	0.007*	0.72*	0.47 ± 0.16	0.36 ± 0.14	0.009*	0.68*
Propofol dose before cessation (mg)	669.74 ± 172.23	647.28 ± 241.68	0.702	0.99	624.39 ± 172.73	566.17 ± 122.36	0.129	0.99
Time to opening eye (min)	11.18 ± 3.32	19.92 ± 5.86	0.001*	1.88*	11.84 ± 3.75	24.71 ± 8.21	0.001*	1.44*
Time to speaking name (min)	13.48 ± 3.54	27.38 ± 7.36	0.001*	1.96*	14.25 ± 3.56	29.71 ± 7.87	0.001*	1.65*
Time to functional mapping (min)	27.11 ± 8.98	37.11 ± 9.54	0.003*	1.14*	25.65 ± 6.69	40.35 ± 10.23	0.001*	1.42*
OAA/S score before mapping	4.96 ± 0.19	4.50 ± 0.78	0.009*	0.36	4.67 ± 0.54	4.19 ± 1.08	0.030*	0.44

Abbreviation: OR, odds ratio; BMI, body mass index; IDH, isocitrate dehydrogenase; KPS, Karnofsky performance scale; FLAIR, fluid-attenuated inversion recovery; Ce, effect-site concentration; OAA/S score, Observer Assessment of Alertness/Sedation Scale; NA, non-available. ^#^*p* value was calculated using the chi-square test for categorical variables and the analysis of variance (ANOVA) for continuous variables. Adjusted OR was calculated by adjusted with age, sex, and BMI. *p < 0.05

### Somnolence during functional mapping and the impact on extent of tumor resection

In this cohort, we identified 11 patients (9.4% of 117 patients) with somnolence (poor cognition with OAA/S score < 4) during functional mapping, and three (2.56%) of them failed to complete functional mapping. These patients with somnolence had a significantly longer time to speaking names (median duration, 34.90 min) and lower GTR (54.54%) than other patients without somnolence. When comparing the extent of tumor resection between patients with delayed and faster awakening, we found that patients with prolonged awakening had significantly lower GTR (52.38% vs. 80.95%, *p* = 0.007) after adjusting for age- and BMI-matched comparisons (Table 4 and Supplementary Fig. 2).

**Table 4:** Comparison of tumor resection rate stratified by time to speaking name, history of craniotomy, and IDH mutation status

**Variables**	**Time to speaking name < 20 min (n = 60)**	**Time to speaking name ≥ 20 min (n = 57)**	***p*** **value**^**#**^
Resection rate (%)			0.001*
Gross total resection (95–100%)	48 (80.00%)	31 (54.39%)	
Subtotal resection (85–95%)	1 (1.67%)	13 (22.81%)	
Partial resection (< 85%)	11 (18.33%)	13 (22.81%)	
**Variables**	**Time to speaking name < 20 min (n = 42)**	**Time to speaking name ≥ 20 min (n = 42)**	***p*** **value**^**#**^
Resection rate (%)	**Age and BMI 1:1 propensity-matched comparison**		0.007*
Gross total resection (95–100%)	34 (80.95%)	22 (52.38%)	
Subtotal resection (85–95%)	1 (2.38%)	9 (21.43%)	
Partial resection (< 85%)	7 (16.67%)	11 (26.19%)	
**Variables**	**First craniotomy (n = 59)**	**Repeated craniotomy (n = 58)**	***p*** **value**^**#**^
Resection rate (%)			0.153
Gross total resection (95–100%)	44 (74.58%)	35 (60.34%)	
Subtotal resection (85–95%)	4 (6.78%)	10 (17.24%)	
Partial resection (< 85%)	11 (18.64%)	13 (22.41%)	
**Variables**	**IDH1 mutation (n = 54)**	**IDH1 wild-type (= 63)**	***p*** **value**^**#**^
Resection rate (%)			0.084
Gross total resection (95–100%)	31 (58.49%)	48 (76.19%)	
Subtotal resection (85–95%)	7 (13.21%)	7 (11.11%)	
Partial resection (< 85%)	15 (28.30%)	8 (12.70%)	

Abbreviation: BMI, body mass index; IDH, isocitrate dehydrogenase. ^#^*p* value was calculated using the chi-square test for categorical variables and the analysis of variance (ANOVA) for continuous variables. *p < 0.05

## Discussion

This retrospective single-center cohort study aimed to identify the predictors of prolonged awakening after propofol cessation during MAC for awake craniotomy. Age- and BMI-matched comparisons showed that patients with prolonged awakening more often had IDH1 wild-type tumors and had undergone repeated craniotomies. Subgroup analyses showed that older age and larger T2/FLAIR volume were associated with prolonged awakening in patients receiving the first craniotomy, while lower preoperative KPS scores and depression were associated with prolonged awakening in patients receiving repeated craniotomies. In patients with IDH1 mutant tumors, a higher WHO grade and repeated craniotomies contributed to delayed awakening. As delayed awakening was associated with somnolence during functional mapping and a lower GTR, the predictors of prolonged awakening identified in our analysis serve as a reminder to carefully adjust the anesthetic regimen for these patients to facilitate functional mapping. For example, the depth of anesthesia monitoring should be utilized to maintain moderate sedation on an individualized basis. Additionally, the use of adjuncts, such as dexmedetomidine, remifentanil, or remimazolam (which could be reversed by flumazenil) may help reduce propofol dosage and expedite emergence [20–24].

In our MAC regimen for awake craniotomy, propofol is the solely used anesthetic before awakening and a propofol Ce of 2.0–2.2 µg/mL is administered in every participant. Propofol induces unconsciousness through inhibition of the GABA type A receptor and induces the breakdown of cortical and subcortical functional connectivity, thus preventing communication between lower-level sensory input and higher-order frontoparietal networks [25]. Therefore, patients with brain tumors located in or near the eloquent cortex may require a longer duration to reconnect these disrupted networks during emergence from propofol sedation [26]. In our analysis, patients with repeated craniotomies and IDH1 wild-type tumors had a delayed time to speaking after propofol cessation, possibly due to greater frontoparietal network or white matter language tract damage [27].

Recurrence of brain tumors is increasingly common due to improved treatments and higher survival rates, particularly in patients with high-grade brain tumors. Re-resection of recurrent gliomas is crucial method to prolong survival, emphasizing the importance of maximizing resection in repeated craniotomies [4, 28]. However, repeated surgery is associated with increased difficulty in identifying remodeled language tracts and a higher risk of perioperative morbidities [27]. We found that patients with lower preoperative KPS scores and depression were more likely to delay awakening during repeated craniotomies. These patients may have experienced a greater extent of neural network destruction during the previous craniotomy, leading to delayed emergence from propofol sedation. Regarding the association between depression and delayed awakening, patients with depression may have functional circuit abnormalities across multiple brain structures and neurotransmission, thus hindering reconnection during recovery from propofol sedation [29].

IDH1 wild-type tumors are often observed in patients with advanced brain tumors and are often associated with larger tumor volume, lower KPS, and worse clinical outcomes than patients with IDH1 mutations [30]. We found that a higher WHO grade and repeated craniotomy contributed to delayed awakening in patients with IDH1 mutant tumors. Although patients with IDH1 mutations generally have less invasive tumor characteristics than patients with wild-type IDH1 tumors, a higher WHO grade and repeated craniotomy might confer extensive neural network destruction, thus contributing to prolonged awakening.

Delayed awakening was associated with somnolence during functional mapping and lower GTR in our analysis. Somnolence during functional mapping had contributed to lower GTR in our cohort (GTR in somnolent vs. non-somnolent patients: 54.54% vs. 77.35%), because somnolence could compromise the identification of the eloquent cortex and make it difficult to minimize an iatrogenic neurological deficit. Our identified predictors to prolonged awakening (repeated craniotomy and IDH wildtype tumor) were not the cause of lower GTR because they both had no significant association with tumor resection rate (Table 4). Further larger cohort using tools such as tractography is required to better clarify the causal relationship between delayed awakening and lower GTR.

To the best of our knowledge, this is the first study to analyze the predictors of delayed awakening using the MAC technique for awake craniotomy. Generally, the MAC technique in awake craniotomy accelerates awakening more efficiently after sedative discontinuation than the SAS technique due to reduced anesthetics requirements for maintaining moderate sedation [17]. The SAS technique for awake craniotomy was utilized in the Japanese and Dutch cohorts, and the analyzed predictors were not applicable to our MAC technique [10, 11]. In the Japanese cohort, 25.5% of patients had somnolence during functional mapping, and 10.9% failed brain mapping [11]. In our cohort, there were fewer somnolent patients (11 patients, 9.40%) and only three (2.56%) failed functional mapping. The Dutch cohort defined the time to wake up as the time from discontinuation of propofol and remifentanil to extubation [10]. Therefore, their predictors such as age and BMI may have a greater association with propofol and opioid pharmacokinetics. Our identified predictors of delayed awakening using solely propofol sedation for MAC after adjusted with age and BMI, might be more strongly correlated with glioma pathology and propofol-related network connectivity [31]. Future studies examining the association between brain tumor functionality using tractography and anesthetic-related network connectivity may further elaborate our findings [27].

This study has several limitations. First, because this was a retrospective study, many demographic differences could not be fully eliminated through statistical adjustments. Second, we applied a similar propofol-maintained Ce for every participant from the skin incision to the dural opening to maintain a modified OAA/S score of 0–2 without individual adjustment. Therefore, some patients might have been oversedated, leading to delayed awakening or somnolence during functional mapping, whereas others might have been undersedated, with faster emergence. Third, the anesthesiologist was not the same for all participants (Dr C.-M. Lin took charge of 95.72% cases); therefore, the detailed anesthesia management and quality of the scalp block may have been different. Fourth, tumor involvement of language/motor pathways was not checked with pre-operative tractography in this cohort because that was unavailable in our institute during the study period. Future prospective grant-supported studies are required to utilize anesthesia depth monitoring to deliver individualized sedation and to incorporate adjuncts such as remifentanil, dexmedetomidine, or remimazolam into the current MAC regimen to enhance emergence [32].

## Conclusion

In this retrospective single-center cohort of patients undergoing MAC for awake craniotomy, we identified patients with IDH1 wild-type tumors and repeated craniotomies were associated with delayed awakening after propofol discontinuation. Patients with delayed awakening had more somnolent episodes during functional mapping and had a lower GTR than those with faster awakening. Therefore, meticulous titration of the anesthetic regimen according to individualized anesthetic depth monitoring should be performed in susceptible patients. Our results have the potential to improve the knowledge of propofol-related functional network connectivity and enhance the recovery of patients undergoing awake craniotomy.

### Electronic supplementary material

Below is the link to the electronic supplementary material.

Supplementary Table 1. Demographics and perioperative variables stratified by time to speaking name. Table 2. Comparison between patients with first or repeated brain tumor surgery. Table 3. Comparison between patients with IDH1 mutant and wild-type tumors. Fig. 1 Scatter plot of tumor volume and time to speaking name in patients with IDH1 wild-type versus IDH1 mutant gliomas. Fig. 2; Comparison of tumor resection rate according to patients with (A) diverse time to speaking name (age- and BMI-matched comparison), (B) IDH1 mutation status, and (C) history of craniotomy.
