# Supplementary material for: Predictors for delayed awakening in adult glioma patients receiving awake craniotomy under monitored anesthesia care
Source: J Neurooncol. 2023 Nov 2;165(2):361–72. doi: 10.1007/s11060-023-04494-1 (PMC10689299; doi:10.1007/s11060-023-04494-1)

**Supplementary Table 1. Demographics and perioperative variables stratiﬁed by time to speaking name**

| **Variables** | **Time to speaking name < 20 min (n=60)** | **Time to speaking name ≥ 20 min (n=57)** | ***p* ^#^** | **Adjusted OR^#^** |
| --- | --- | --- | --- | --- |
| Age (mean±SD, years) | 47.75±15.37 | 52.21±13.81 | 0.102 | 1.02 |
| Male sex, N(%) | 33 (55.00%) | 25 (47.61%) | 0.666 | 0.83 |
| BMI (kg/m^2^) | 25.44±4.38 | 23.91±3.26 | 0.034* | 0.90* |
| Preoperative creatinine (mg/dL) | 0.73±0.19 | 0.69±0.18 | 0.262 | 0.69 |
| Preoperative total bilirubin(mg/dL) | 0.53±0.22 | 0.55±0.21 | 0.798 | 0.91 |
| Preoperative hypnotics use | 5 (8.47 %) | 3 (5.26%) | 1.000 | 0.67 |
| Preoperative KPS score | 88.07±8.95 | 83.52±10.84 | 0.017* | 0.94* |
| Hypertension | 17 (28.33%) | 10 (17.54%) | 0.166 | 0.54 |
| obesity | 9 (15.00%) | 3 (5.26%) | 0.315 | 0.41 |
| Diabetes | 5 (8.33%) | 3 (5.26%) | 0.717 | 0.61 |
| Old stroke | 1 (1.67%) | 3 (5.26%) | 0.356 | 3.28 |
| smoking | 10 (16.67%) | 4 (7.02%) | 0.108 | 0.37 |
| History of brain tumor surgery | 17 (38.63%) | 26 (61.90%) | 0.031* | 2.96* |
| Neuropsychological evaluation |  |  |  |  |
| Aphasia | 15 (25.86%) | 17 (32.69%) | 0.431 | 1.39 |
| Depression | 12 (21.05%) | 16 (30.76%) | 0.246 | 1.67 |
| Executive dysfunction | 16 (28.07%) | 22 (42.31%) | 0.119 | 1.87 |
| Brain tumor characters |  |  |  |  |
| Left hemisphere | 36 (60.00%) | 38 (66.67%) | 0.779 | 1.11 |
| Right hemisphere | 24 (40.00%) | 19 (33.33%) |  | 0.82 |
| Frontal lobe | 38 (63.33%) | 32 (56.14%) | 0.934 | 1.00 |
| Temporal lobe | 13 (21.67%) | 14 (24.56%) | 0.934 | 1.15 |
| Parietal lobe | 5 (8.33%) | 6 (10.52%) | 0.934 | 3.18 |
| Corpus callosum | 18 (30.00%) | 12 (21.05%) | 0.934 | 0.62 |
| Insula | 3 (5.00%) | 3 (5.26%) | 0.934 | 0.99 |
| Hippocampus | 1 (1.67%) | 0 (0%) | 0.934 | NA |
| T1 enhanced volume (ml) | 8.56±14.67 | 12.75±18.14 | 0.243 | 1.02 |
| T2/FLAIR volume (ml) | 79.27±74.11 | 86.79±56.93 | 0.603 | 1.00 |
| Midline shift(mm) | 3.47±4.56 | 4.13±8.25 | 0.601 | 1.02 |
| WHO grade | 2.93±0.95 | 3.31±0.71 | 0.041* | 1.41* |
| IDH1 mutation | 27 (45.76%) | 26 (45.61%) | 0.743 | 0.99 |
| Propofol Ce (opening eyes) (μg/mL) | 0.79±0.19 | 0.56±0.25 | <0.001* | 0.01* |
| Propofol Ce (speaking name) (μg/mL) | 0.72±0.19 | 0.48±0.17 | <0.001* | 0.01* |
| Propofol dose before cessation (mg) | 646.39±171.32 | 601.56±186.67 | 0.182 | 0.99 |
| Time to opening eye (min) | 11.54±3.53 | 22.53±7.57 | <0.001* | 1.54* |
| Time to speaking name (min) | 13.95±3.53 | 28.65±7.66 | <0.001* | 1.54* |
| Time to functional mapping (min) | 26.48±7.82 | 38.87±9.96 | <0.001* | 1.21* |
| OAA/S score before mapping | 4.78±0.47 | 4.25±1.03 | 0.004* | 0.39* |
| Somnolence | 1 (1.67%) | 10 (17.54%) | 0.003* | 0.09* |

Abbreviation: OR, odds ratio; BMI, body mass index; IDH, isocitrate dehydrogenase; KPS, Karnofsky performance scale; FLAIR, Fluid attenuated inversion recovery; Ce, effect-site concentration; OAA/S score, Observer Assessment of Alertness/Sedation Scale; NA, non-available. ^#^ *p* value was calculated using the chi-square test for categorical variables and the analysis of variance (ANOVA) for continuous variables. Adjusted OR was calculated by adjusted with age, sex, and BMI. **p* < 0.05.

**Supplementary Table 2. Comparison between patients with first or repeated brain tumor surgery**

| **Variables** | **First brain tumor surgery (n=59)** | **Repeated brain tumor surgery (n=58)** | ***p*^#^** | **Adjusted OR^#^** |
| --- | --- | --- | --- | --- |
| Age (mean±SD, years) | 52.35±16.79 | 47.44±11.93 | 0.071 | 0.97 |
| Male sex, N(%) | 24 (40.68%) | 34 (58.62%) | 0.062 | 1.46 |
| BMI (kg/m^2^) | 24.96±4.15 | 24.42±3.72 | 0.457 | 0.96 |
| Preoperative creatinine (mg/dL) | 0.70±0.19 | 0.71±0.17 | 0.775 | 0.98 |
| Preoperative total bilirubin(mg/dL) | 0.51±0.25 | 0.57±0.19 | 0.449 | 0.86 |
| Preoperative hypnotics use | 4 (6.78%) | 4 (6.89%) | 0.872 | 1.00 |
| Preoperative KPS score | 86.32±8.15 | 85.37±11.93 | 0.629 | 0.99 |
| Hypertension | 12 (20.34%) | 15 (25.86%) | 0.478 | 1.36 |
| Diabetes | 4 (6.78%) | 4 (6.89%) | 0.872 | 1.01 |
| Old stroke | 2 (3.38%) | 2 (3.34%) | 0.954 | 1.01 |
| smoking | 4 (9.30%) | 4 (9.30%) | 1.000 | 1.00 |
| Neuropsychological evaluation |  |  |  |  |
| Aphasia | 17 (30.36%) | 15 (27.77%) | 0.766 | 0.88 |
| Depression | 14 (25.00%) | 14 (26.41%) | 0.866 | 1.07 |
| Executive dysfunction | 19 (33.92%) | 19 (35.84%) | 0.833 | 1.08 |
| Brain tumor characters |  |  |  |  |
| Left hemisphere | 37 (62.71%) | 37 (63.79%) | 0.505 | 0.98 |
| Frontal lobe | 34 (57.62%) | 36 (62.07%) | 0.637 | 0.91 |
| Temporal lobe | 16 (27.11%) | 11 (18.96%) | 0.637 | 1.42 |
| Parietal lobe | 6 (10.16%) | 5 (8.62%) | 0.637 | 1.17 |
| Corpus callosum | 18 (30.51%) | 12 (20.69%) | 0.637 | 0.59 |
| Insula | 2 (3.39%) | 4 (6.89%) | 0.637 | 0.35 |
| Hippocampus | 1 (1.69%) | 0 (0%) | 0.637 | NA |
| T1 enhanced volume (ml) | 17.37±25.25 | 12.59±21.19 | 0.272 | 0.99 |
| T2/FLAIR volume (ml) | 89.68±62.04 | 71.69±69.11 | 0.141 | 1.25 |
| Midline shift(mm) | 5.39±8.24 | 2.12±3.62 | 0.006* | 0.86* |
| WHO grade | 3.18±0.92 | 3.15±0.81 | 0.845 | 1.08 |
| IDH1 mutation | 24 (41.38%) | 29 (50.00%) | 0.351 | 1.41 |
| Ki-67 (%) | 22.57±20.96 | 17.30±18.42 | 0.211 | 0.96 |
| Propofol Ce (opening eyes) (μg/mL) | 0.66±0.26 | 0.69±0.25 | 0.612 | 1.46 |
| Propofol Ce (speaking name) (μg/mL) | 0.59±0.22 | 0.61±0.21 | 0.645 | 1.48 |
| Propofol Ce (mapping) (μg/mL) | 0.44±0.18 | 0.46±0.15 | 0.431 | 1.26 |
| Propofol dose before cessation (mg) | 590.47±129.75 | 658.32±222.52 | 0.053 | 1.01 |
| Time to opening eye (min) | 15.98±8.08 | 17.77±7.96 | 0.229 | 1.02 |
| Time to speaking name (min) | 19.45±8.97 | 22.79±9.68 | 0.055 | 1.02 |
| Time to functional mapping (min) | 31.32±11.06 | 33.74±10.58 | 0.229 | 1.04 |
| OAA/S score before mapping | 4.62±0.74 | 4.52±0.81 | 0.525 | 1.02 |
| Somnolence | 5 (8.62%) | 6 (10.91%) | 0.682 | 0.79 |

Abbreviation: OR, odds ratio; BMI, body mass index; IDH, isocitrate dehydrogenase; KPS, Karnofsky performance scale; FLAIR, fluid-attenuated inversion recovery; Ce, effect-site concentration; OAA/S score, Observer Assessment of Alertness/Sedation Scale; NA, non-available. ^#^ *p* value was calculated using the chi-square test for categorical variables and the analysis of variance (ANOVA) for continuous variables. Adjusted OR was calculated by adjusted with age, sex, and BMI. **p* < 0.05.

**Supplementary Table 3. Comparison between patients with IDH1 mutant and wild-type tumors**

| **Variables** | **IDH1 wild-type (n=63)** | **IDH1 mutation (n=54)** | ***p* ^#^** | **Adjusted OR ^#^** |
| --- | --- | --- | --- | --- |
| Age (mean±SD, years) | 55.98±14.59 | 42.91±11.57 | <0.001* | NA |
| Male sex, N(%) | 30(47.62%) | 27 (50.00%) | 0.721 | NA |
| BMI (kg/m^2^) | 23.91±4.04 | 25.53±3.61 | 0.023* | NA |
| Preoperative creatinine (mg/dL) | 0.69±0.19 | 0.70±0.18 | 0.695 | 0.435 |
| Preoperative total bilirubin(mg/dL) | 0.49±0.24 | 0.61±0.15 | 0.199 | 1.494 |
| Preoperative hypnotics use | 5 (7.93%) | 3 (5.55%) | 0.727 | 0.710 |
| Preoperative KPS score | 83.61±10.49 | 88.57±9.13 | 0.010* | 1.009 |
| Hypertension | 17 (26.98%) | 9 (16.67%) | 0.198 | 0.917 |
| obesity | 6 (9.52%) | 7 (12.96%) | 0.531 | 1.445 |
| Diabetes | 6 (9.52%) | 1 (1.85%) | 0.123 | 0.636 |
| Old stroke | 3 (4.76%) | 1 (1.85%) | 0.624 | 0.385 |
| History of brain tumor surgery | 29 (46.03%) | 29 (53.70%) | 0.351 | 1.276 |
| Neuropsychological evaluation | | | | |
| Aphasia | 21 (36.84%) | 11 (20.37%) | 0.072 | 0.460 |
| Depression | 15 (26.78%) | 13 (24.07%) | 0.832 | 0.911 |
| Executive dysfunction | 22 (39.28%) | 15 (27.77%) | 0.253 | 0.626 |
| Brain tumor characters | | | | |
| Right hemisphere | 22 (34.92%) | 22 (40.74%) | 0.357 | 1.242 |
| Frontal lobe | 30 (47.62%) | 40 (74.07%) | 0.006* | 1.000 |
| Temporal lobe | 19 (30.15%) | 8 (14.81%) |  | 0.275 |
| Parietal lobe | 9 (14.28%) | 1 (1.85%) |  | 0.239 |
| Corpus callosum | 13 (20.63%) | 17(31.48%) |  | 1.579 |
| Insula | 3 (4.76%) | 3 (5.66%) | 0.006* | 1.102 |
| Hippocampus | 1 (1.58%) | 0 (0%) | 0.006* | NA |
| T1 enhanced volume (ml) | 20.20±22.42 | 8.37±12.89 | 0.006* | 0.973* |
| T2/FLAIR volume (ml) | 83.57±68.91 | 76.57±63.11 | 0.573 | 0.998 |
| Midline shift(mm) | 4.19±8.04 | 3.23±4.28 | 0.415 | 0.977 |
| WHO grade | 3.54±0.87 | 2.73±0.62 | <0.001* | 0.386* |
| Ki-67 (%) | 24.86 ± 22.29 | 14.82 ± 15.52 | 0.013* | 0.971* |
| Jafari classification | 2.35 ± 0.98 | 1.87 ± 0.99 | 0.010* | 0.675 |
| Propofol Ce (opening eyes) (μg/mL) | 0.62±0.24 | 0.74±0.25 | 0.007* | 1.605 |
| Propofol Ce (speaking name) (μg/mL) | 0.56±0.21 | 0.66±0.22 | 0.013* | 2.272* |
| Propofol dose before cessation (mg) | 595.74±151.74 | 659.17±205.96 | 0.070 | 1.002 |
| Time to opening eye (min) | 18.17±9.04 | 15.47±6.44 | 0.063 | 0.956 |
| Time to speaking name (min) | 21.85 ± 9.85 | 20.30 ± 9.03 | 0.381 | 0.982 |
| Time to functional mapping (min) | 32.89±11.31 | 32.01±10.46 | 0.670 | 0.992 |
| OAA/S score before mapping | 4.43±0.88 | 4.74±0.59 | 0.032* | 1.227 |
| Somnolence | 7 (11.29%) | 4 (7.41%) | 0.752 | 1.546 |

Abbreviation: OR, odds ratio; BMI, body mass index; IDH, isocitrate dehydrogenase; KPS, Karnofsky performance scale; FLAIR, fluid-attenuated inversion recovery; Ce, effect-site concentration; OAA/S score, Observer Assessment of Alertness/Sedation Scale; NA, non-available. ^#^OR was adjusted by age, sex, and BMI. *p* value was calculated using the chi-square test for categorical variables and the analysis of variance (ANOVA) for continuous variables. Adjusted OR was calculated by adjusted with age, sex, and BMI. **p* < 0.05.

**Supplementary Figure 1.** Scatter plot of tumor volume and time to speaking name in patients with IDH1 wild-type versus IDH1 mutant gliomas.

**
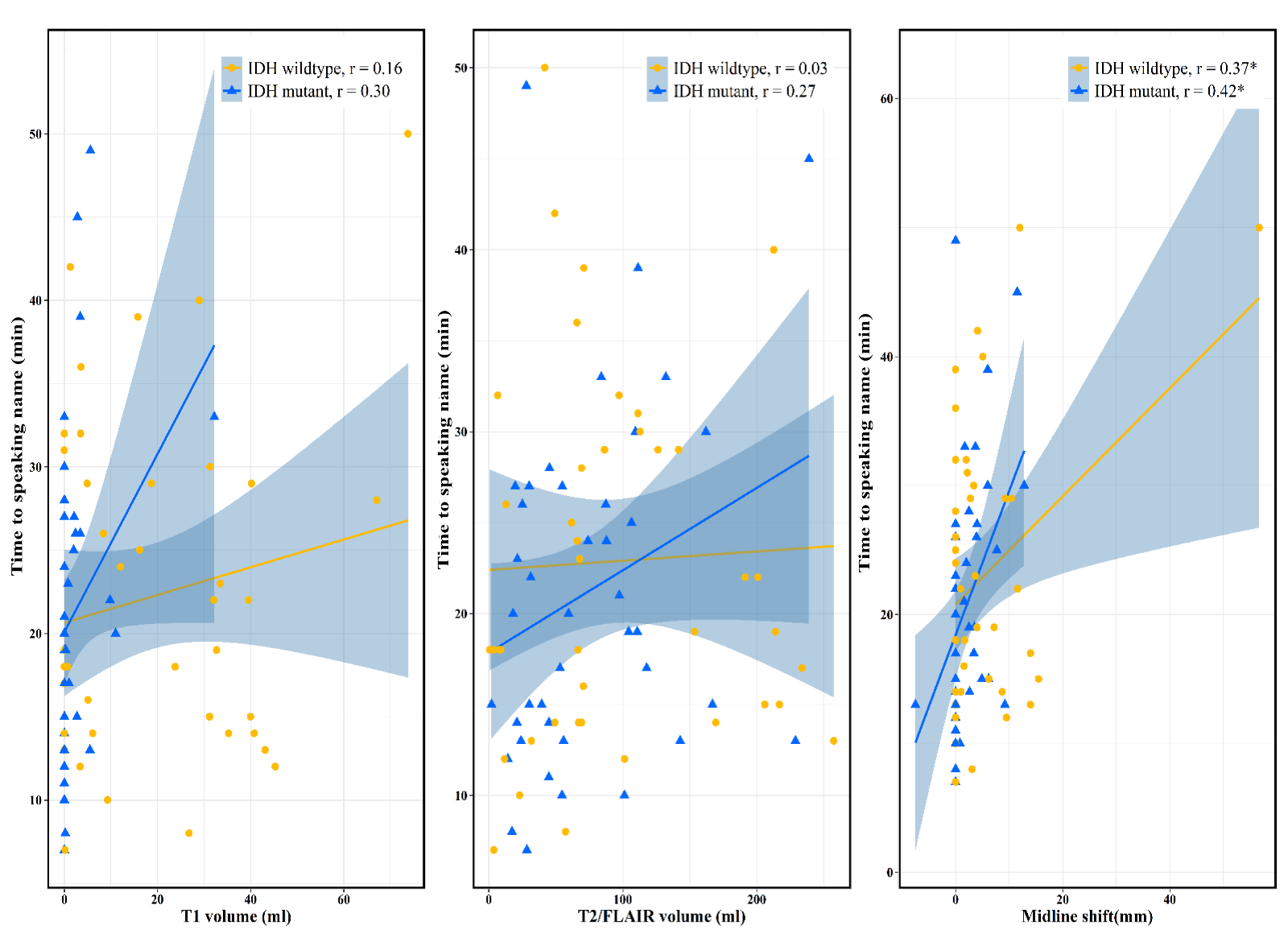
**

**Supplementary Figure 2.** Comparison of tumor resection rate according to patients with (A) diverse time to speaking name (age- and BMI-matched comparison), (B) IDH1 mutation status, and (C) history of craniotomy.


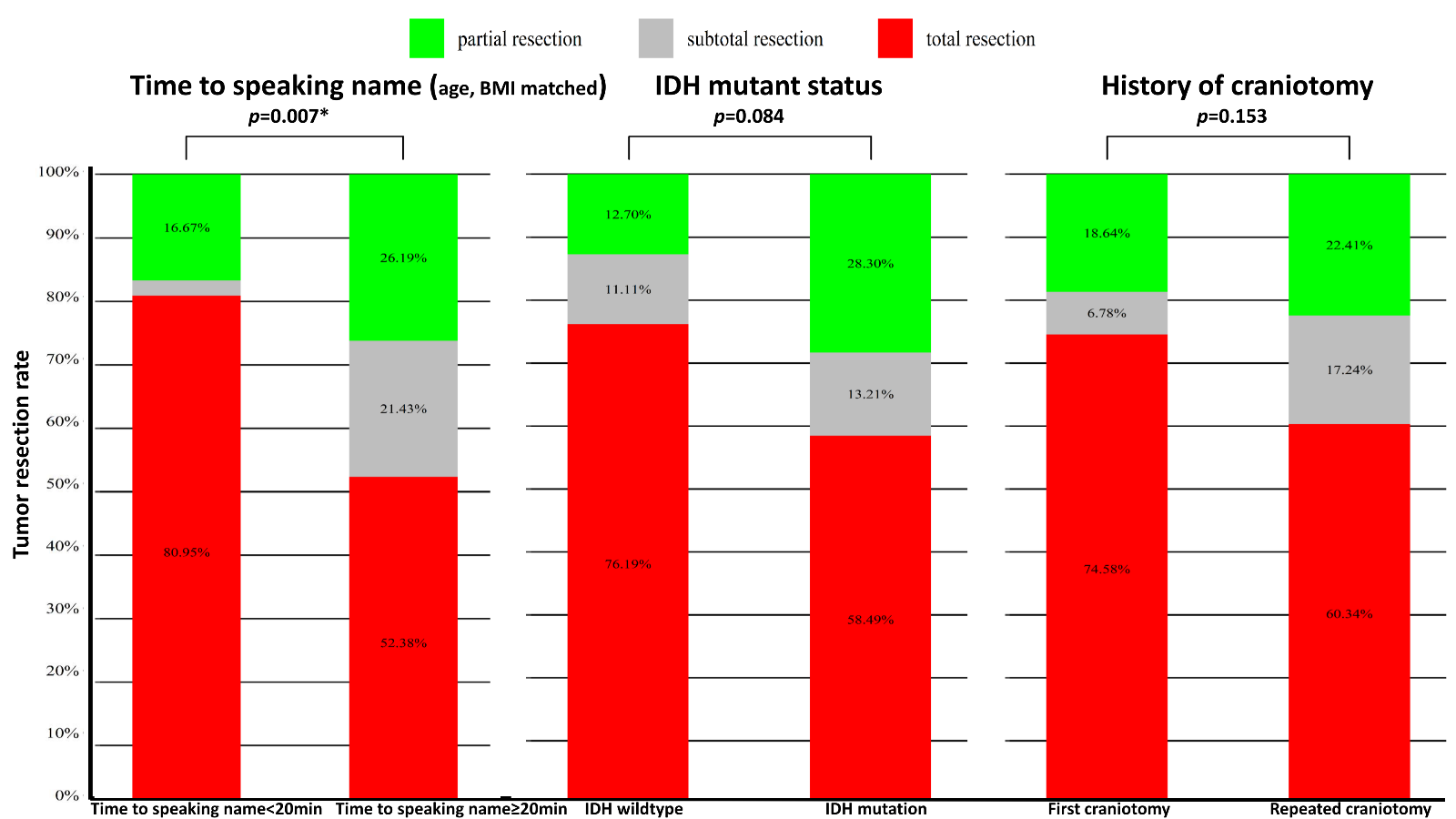

Supplement: Supplementary file 1 — Supplementary Table 1. Demographics and perioperative variables stratified by time to speaking name. Table 2. Comparison between patients with first or repeated brain tumor surgery. Table 3. Comparison between patients with IDH1 mutant and wild-type tumors. Fig. 1 Scatter plot of tumor volume and time to speaking name in patients with IDH1 wild-type versus IDH1 mutant gliomas. Fig. 2; Comparison of tumor resection rate according to patients with (A) diverse time to speaking name (age- and BMI-matched comparison), (B) IDH1 mutation status, and (C) history of craniotomy. [file 11060_2023_4494_MOESM1_ESM.docx]
